# Accelerated Biological Aging, Genetic Susceptibility, and Non-Alcoholic Fatty Liver Disease: Two Prospective Cohort Studies

**DOI:** 10.3390/nu17101618

**Published:** 2025-05-08

**Authors:** Ying Zhao, Yu Wang, Li Chen, Huimin Chen, Yuhan Tang, Yuefeng He, Ping Yao

**Affiliations:** 1 School of Public Health, Kunming Medical University, Kunming 650500, China; zhaoying1@kmmu.edu.cn; 2 Department of Nutrition and Food Hygiene, Hubei Key Laboratory of Food Nutrition and Safety, Ministry of Education Key Laboratory of Environment and Health and MOE Key Lab of Environment and Health, Key Laboratory of Environment and Health (Wuhan), Ministry of Environmental Protection, State Key Laboratory of Environment Health (Incubation), School of Public Health, Tongji Medical College, Huazhong University of Science and Technology, Wuhan 430030, China; wy128137@163.com (Y.W.); d202081547@hust.edu.cn (H.C.); 2015220157@hust.edu.cn (Y.T.); 3 Hubei Key Laboratory of Lipid Chemistry and Nutrition, and Key Laboratory of Oilseeds Processing, Ministry of Agriculture, Oil Crops and Lipids Process Technology National & Local Joint Engineering Laboratory, Oil Crops Research Institute of the Chinese Academy of Agricultural Sciences, Wuhan 430062, China; chenli05@caas.cn

**Keywords:** biological aging, KDMAge, PhenoAge, non-alcoholic fatty liver disease, polygenic risk score, genetic susceptibility

## Abstract

**Background**: Biological aging is considered a vital risk factor for age-related diseases, but its role in non-alcoholic fatty liver disease (NAFLD) remains uncertain. This study aimed to evaluate the associations of biological aging with NAFLD and the modified effect of genetic susceptibility. **Methods**: This study included 329,040 participants from the UK Biobank and 6783 participants from the Dongfeng–Tongji Cohort in China. We calculated the chronological age-adjusted biological age as a surrogate measure for biological aging. Accelerated aging was defined as biological age that exceeded chronological age. The association between biological aging and the risk of NAFLD was assessed in the two cohorts. Polygenic risk scores (PRSs) were used to determine genetic susceptibility for NAFLD in the UK Biobank and further analyze the interaction with biological aging. **Results**: In the UK Biobank, one year older in age-adjusted biological age increased prevalent NAFLD risk by 6%. The hazard ratios (HRs) and 95% confidence intervals (95% CIs) of NAFLD by accelerated aging were 1.35 (1.17, 1.56) and 1.69 (1.54, 1.85) compared to non-aging. In the Dongfeng–Tongji Cohort, biological aging was prospectively associated with NAFLD (accelerated aging: odds ratio (OR) (95% CI) = 1.18 (1.03, 1.36)). In the UK Biobank, high genetic risk was significantly associated with higher NAFLD risk compared to low genetic risk (HRs (95% CIs) = 1.65 (1.40, 1.95)). Analyses of joint effects showed that participants with high PRS and accelerated aging had the highest risk of NAFLD [2.66 (2.98, 3.57) and 2.06 (2.36, 3.96)]. However, biological aging was prospectively associated with NAFLD among participants regardless of genetic risk. There was no significant interaction between genetic risk and biological aging. **Conclusions**: Accelerated biological aging was associated with a higher risk of NAFLD independent of genetic susceptibility. Identifying populations with accelerated biological aging by the use of surrogate measures and timely intervention may be beneficial for the prevention of NAFLD.

## 1. Introduction

Non-alcoholic fatty liver disease (NAFLD), a major cause of cirrhosis and hepatocellular carcinoma, affects 25% of the global population [1]. In addition to the growing incidence, NAFLD characterized by excessive hepatic fat accumulation has attracted more attention because of its direct association with metabolic risk factors like insulin resistance and various chronic metabolism diseases with heavy burden [2]. In 2016, estimates indicated that in the United States, more than 64 million individuals suffered from NAFLD. The annual direct medical expenses associated with this reached approximately USD 103 billion. In France, Germany, Italy, and the UK, around 52 million individuals had NAFLD, incurring an annual cost of roughly EUR 35 billion [3]. Given that no medical treatment for NAFLD has been clinically approved thus far, pinpointing newly emerging risk factors for this disorder could potentially aid in formulating prevention or treatment approaches [4].

As a central metabolic organ, the liver plays a primary role in age-associated imbalances of glucose homeostasis, lipid processing, and detoxification pathways [5,6]. The incidence of NAFLD follows a pattern that aligns with age [3]. NAFLD chiefly impacts middle-aged and elderly individuals since associated risk factors typically rise as age increases [7]. Additionally, older patients often demonstrate more pronounced biochemical, hematological, and histological alterations [8]. Previous animal studies have shown that aging may be a pathological mechanism of NAFLD, but this has not been validated in the population [9,10].

Additionally, people of the same chronological age exhibit varying aging rates and different susceptibilities to age-related diseases [11]. Biological age, measured by composite biomarkers such as KDMAge and PhenoAge, offers a more comprehensive assessment of an individual’s biological aging status compared to chronological age alone. KDMAge is calculated using multiple clinical biomarkers including total cholesterol, forced expiratory volume in one second, systolic blood pressure, albumin, and others. PhenoAge is derived from a set of nine aging-related variables. Detecting individuals whose biological age exceeds their chronological age, even in the absence of obvious health problems, could help implement timely interventions to prevent disease. Thus, we want to figure out whether biological aging can serve as an indicator for predicting the incident risk of NAFLD. Previous studies focused on single aging markers (e.g., telomere length or DNA methylation age) [12,13], with no prospective cohort studies examining the role of composite biological age metrics in NAFLD incidence. To address this gap, our study evaluated prospective associations between biological aging and NAFLD using data from two ethnically distinct cohorts.

Genetic susceptibility is crucial in the pathogenesis of NAFLD [14], and a multitude of prior genome-wide association studies (GWASs) have pinpointed numerous single-nucleotide polymorphisms (SNPs) linked to NAFLD [15,16]. Recently, the outcomes of a large-scale GWAS were made public. This GWAS focused on chronically elevated serum alanine aminotransferase levels (ALTs) (*n* = 218,595), intrahepatic lipid content evaluated by imaging (*n* = 44,289), and biopsy-verified NAFLD (*n* = 63,969) [17]. These data enable the exploration of polygenic risk scores (PRSs) and the combined effects of multiple risk factors to identify individuals at potential risk for NAFLD.

The current study is the first to simultaneously analyze data from two large-scale prospective cohorts, the UK Biobank and the Dongfeng–Tongji Cohort. The participants in these two cohorts are from different ethnic backgrounds. This enables us to systematically explore the effect of biological aging on the risk of NAFLD. Moreover, we further explored whether this association exists regardless of genetic risk.

## 2. Methods

### 2.1. Study Design and Population

The UK Biobank recruited more than 500,000 participants from across the UK between 2006 and 2010, gathering comprehensive phenotype and genotype data [18]. More details about the UK Biobank protocol can be found online (http://www.ukbiobank.ac.uk, accessed on 1 January 2024). The UK Biobank was approved by the North West Research Ethics Committee in 2006 (06/MRE08/65) and has been renewed every five years since then. All participants provided written informed consent. We made a specific request to the UK Biobank (application number 88159) to gain access to the data required for this study. All participants gave written informed consent before enrollment in this study. In the UK Biobank, we excluded participants missing information on biological age at baseline (*n* = 170,158). We also excluded participants who had NAFLD or other liver disease at baseline (*n* = 1910). In addition, we excluded participants whose data of NAFLD diagnosis were deficient (*n* = 45). Moreover, participants who used medicine with known hepatotoxicity were further excluded (*n* = 1248). Finally, a total of 329,040 participants were included in the current analysis (Figure S1).

The Dongfeng–Tongji Cohort was initiated in 2008 among retirees of the Dongfeng Motor Corporation (DMC) in Shiyan City, Hubei Province, China [19]. During the enrollment window (September 2008–June 2010), 27,009 individuals (87% of 31,000 invited retirees) completed baseline assessments. Ethical approval was obtained from both Tongji Medical College and DMC Hospital Ethics Committees in 2008 prior to the baseline survey. Written informed consent was universally obtained. From the initial cohort (*n* = 27,009), participants lacking KDMAge data (*n* = 7757) were preliminarily excluded. In addition, subjects at baseline with the presence of any of following conditions were excluded: chronic hepatitis (*n* = 709), hepatic cirrhosis (*n* = 11), fatty liver disease (*n* = 7122), B-ultrasonography information deficiency (*n* = 60), excessive alcohol consumption (*n* = 645), and usage of medications with known hepatotoxicity (*n* = 101). Subsequently, individuals with missing information on outcome (*n* = 3821) were further excluded from the 10,604 participants included in the follow-up study. Finally, the eligible sample size for analyses was 6783 in the present study (Figure S2).

### 2.2. Outcomes

In the UK Biobank, NAFLD was determined based on cases of hospitalization or death directly linked to NAFLD or NASH. Death dates and causes were sourced from death certificates maintained by the National Health Service (NHS) Information Centre for England and Wales and the NHS Central Register Scotland for Scotland. According to the International Classification of Diseases, 10th revision (ICD-10), and the latest Expert Panel Consensus Statement [18], NAFLD was categorized as ICD-10 K76.0 (fatty [change of] liver, not elsewhere classified) and K75.8 (NASH, other specified inflammatory liver diseases). The UK Biobank has specific onset times for NAFLD cases and is currently under ongoing follow-up, with a median follow-up time of 12.45 years.

In the Dongfeng–Tongji Cohort, each participant underwent a single high-sensitivity ultrasound examination conducted by a skilled radiologist using an Aplio XG (TOSHIBA, Tokyo, Japan) machine to assess NAFLD at baseline. Hepatic steatosis was diagnosed if at least two of the following three abnormalities were observed during abdominal ultrasonography: a hepatorenal or hepatosplenic echogenicity gradient (liver parenchyma brighter than kidney/spleen) [20]. To exclude secondary causes of fatty liver, participants were required to meet stringent inclusion criteria: a daily alcohol intake <30 g (male) or <20 g (female), absence of HBV infection confirmed by serological testing, no clinical or imaging evidence of chronic hepatitis/cirrhosis, and no recent use (within 14 days) of steatosis-inducing medications (including tamoxifen, amiodarone, and valproate). The participants in the Dongfeng–Tongji Cohort were followed up for 10 years.

### 2.3. Assessment of Biological Aging

In the UK Biobank, the assessment of biological aging involved biological age metrics, namely KDMAge and PhenoAge. KDMAge, put forward by Klemera and Doubal [21], was calculated based on clinical biomarkers, including total cholesterol, forced expiratory volume in one second, systolic blood pressure, albumin, blood urea nitrogen, creatinine, C-reactive protein, glycated hemoglobin, and alkaline phosphatase [22]. PhenoAge was derived from 10 aging-related variables, as outlined in a previously published method [23]. The variables encompassed chronological age, red blood cell distribution width, albumin, white blood cell count, lymphocyte percent, glucose, mean cell volume, creatinine, alkaline phosphatase, and C-reactive protein. The values of biological age were computed with the help of the “BioAge” package under R 4.4.0 (https://github.com/dayoonkwon/BioAge, accessed on 1 January 2024).

In the Dongfeng–Tongji Cohort, 10 features were utilized for KDMAge calculation, as outlined in prior research: blood glucose, creatinine, systolic blood pressure, serum urea nitrogen, mean corpuscular hemoglobin, uric acid, platelets, neutrophils, lymphocyte percentage, and direct bilirubin [24]. In brief, the KDM algorithm combines data from multiple regressions of chronological age (CA) on each feature to generate a composite biological age (BA) measure, which shares the same unit as CA.

To assess biological aging, KDMAge and PhenoAge were regressed against chronological ages at the time of biomarker measurement, and residual values were calculated, referred to as KDMAge/PhenoAge acceleration or age-adjusted biological age. Participants with KDMAge/PhenoAge acceleration values greater than 0 were classified as having accelerated aging, while those with values below 0 were categorized as non-aging.

### 2.4. Covariates

All analyses adjusted covariates for age (continuous), sex (male or female), body mass index (<24, 24–28, and ≥28 for the Chinese population; <25, 25–30, and ≥30 for the UK population), ethnicity (White or non-White in the UK Biobank), smoking status (never, ever, or current), alcohol intake (never, ever, or current), education level (less than high school or high school or above), and physical activity (assessed by the metabolic equivalent task based on the International Physical Activity Questionnaire (IPAQ)).

### 2.5. PRS Calculation of NAFLD

The PRS for NAFLD was constructed using SNPs (Table S7) from a genome-wide association study [17], weighted by the multivariable-adjusted risk estimates (β coefficients) for NAFLD: PRS $=\sum_{i=1}^{43}\beta i\times SNPi$.

In this study, the PRS was divided into three categories: low risk (lowest quintile), intermediate risk (quintiles 2 to 4), and high risk (highest quintile) [25].

### 2.6. Statistical Analysis

Baseline characteristics were described using means (standard deviations) or medians (interquartile ranges) for continuous variables and percentages for categorical variables.

Biological aging was evaluated using the following methods: Firstly, KDMAge/PhenoAge acceleration was either treated as a continuous variable or divided into four quartiles (Q1, Q2, Q3, and Q4). Second, participants were classified into two groups based on their accelerated aging status, with those having a KDMAge/PhenoAge acceleration value greater than 0 categorized as “Accelerated aging” and those with a value less than 0 categorized as “Non-aging”. Cox proportional hazards models were used to estimate the hazard ratios (HRs) and corresponding 95% confidence interval (CI) for the associations of biological aging with the risk of incident NAFLD. Schoenfeld residuals were used to assess the proportional hazards assumption. Logistic regression was applied to estimate the association between biological aging and the risk of NAFLD in the Dongfeng–Tongji Cohort. The trend test across increasing exposure groups was performed using integer values (1, 2, 3, and 4). The dose–response relationship between biological aging and NAFLD risk was evaluated using restricted cubic spline regression with three knots at the 25th, 50th, and 75th percentiles.

Additionally, potential modifications with NAFLD, such as age (<65 and ≥65 years), sex (male or female), race (non-White and White), education level (less than high school or high school or above), body mass index (BMI, <24/25, 24–28/25–30, or ≥28/30 kg/m^2^), smoking status (never, ever, or current), and alcohol intake (never, ever, or current), were examined by stratified analyses and interaction testing.

We performed a series of sensitivity analyses to assess the robustness of the findings: (1) excluding the subjects with less than 2 years of follow-up in the UK Biobank; (2) further adjusting for liver-disease-related covariates in the two cohorts (glutamic-pyruvic transaminase (ALT), glutamic oxaloacetic transaminase (AST), and total bilirubin (TBIL)); (3) filling in the missing values in the two cohorts, where missing data for covariates were treated by assigning a missing indicator category for categorical variables and imputing the median for continuous variables; (4) further adjusting the participants’ illness status in the two cohorts (hypertension, diabetes, hyperlipidemia, and cancer).

## 3. Results

### 3.1. Baseline Characteristics of Participants

Table 1 shows the baseline characteristics of participants by non-NAFLD or NAFLD from the UK Biobank and Dongfeng–Tongji Cohort. Among the 329,040 participants from the UK Biobank (mean age 56.4 years, 53.9% female), 3774 incident NAFLD cases were documented. Among the 6783 participants from the Dongfeng–Tongji Cohort (mean age 62.0 years, 58.5% female), 1798 NAFLD cases were documented. In the UK Biobank, participants with NAFLD were older, had lower education levels, and were more likely to be male, non-White, smokers, non-drinkers, and obese. They also had a lower metabolic equivalent task. NAFLD patients may exhibit higher biological age compared to non-NAFLD participants. In the Dongfeng–Tongji Cohort, participants with NAFLD were younger and were more likely to be female, non-smokers, non-drinkers, and obese. They also had a lower metabolic equivalent task. NAFLD patients may exhibit higher biological age compared to non-NAFLD participants.

### 3.2. Association of Biological Aging with NAFLD

**Figure 1:**
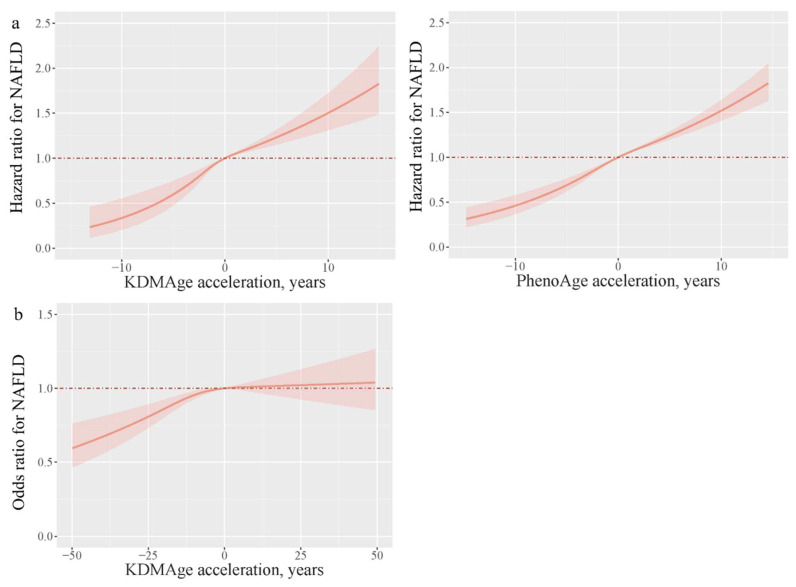
Dose–response relationships of KDMAge/PhenoAge acceleration with the risk of prevalent and incident NAFLD assessed by restricted cubic spline regression ((**a**), UK Biobank; (**b**), Dongfeng–Tongji Cohort). Models were adjusted for age, sex, body mass index, ethnicity (in the UK Biobank), smoking status, alcohol intake, metabolic equivalent task, and education level. The lines and shaded areas represent the odds ratio and the 95% CI.

Both KDMAge acceleration and PhenoAge acceleration showed a positive association with the risk of developing NAFLD; the HRs (95% CI) were 1.06 (1.04, 1.07) and 1.06 (1.05, 1.06), respectively, in the UK Biobank. Accelerated aging was also associated with an elevated NAFLD risk [KDMAge: 1.3 (1.17, 1.56); PhenoAge: (1.69 (1.54, 1.85))] compared to non-aging. Consistent results were reproduced in the quantile analyses (all *p* for trend < 0.001) (Table 2). The dose–response analysis indicated that both KDMAge acceleration and PhenoAge acceleration exhibited a linear relationship with the risk of developing NAFLD in the UK Biobank (Figure 1). In the Dongfeng–Tongji Cohort, the OR (95% CI) for KDMAge acceleration was 1.01 (1.00, 1.01). Compared to non-aging individuals, accelerated aging individuals also exhibited an increased risk of NAFLD [KDMAge: 1.18 (1.03, 1.36)]. Quantile analyses obtained consistent results (*p* for trend <0.001) (Table 2). In the Dongfeng–Tongji Cohort, KDMAge acceleration did not show a linear relationship with the risk of developing NAFLD (Figure 1).

To test the robustness of the association, we performed a series of sensitivity analyses. After excluding participants who developed outcomes within the first 2 years of follow-up in the UK Biobank (Table S1), further adjusting liver-disease-related indicators such as ALT, AST, and TBIL in the two cohorts, imputing missing data in the two cohorts, or further adjusting the participant’s illness status in the two cohorts (hypertension, diabetes, hyperlipidemia, and cancer), the results were not substantially different (Tables S2–S4).

Figure S3 shows the results stratified by sex, age group, race (in the UK Biobank), BMI group, smoking status, alcohol intake, and education level. In the UK Biobank, the positive relations of KDMAge/PhenoAge acceleration and accelerated aging with NAFLD were significantly stronger in participants that were current drinkers (vs. non-drinkers). There were significant interactions of KDMAge with BMI, smoking status, and sex on the risk of NAFLD (all *p* for interaction < 0.05). In the Dongfeng–Tongji Cohort, the positive relations of KDMAge acceleration with NAFLD were significantly stronger in participants that were female (vs. male), younger (vs. those older), and non-smokers (vs. current smokers) and in participants with lower education levels (vs. those with higher education levels).

### 3.3. Risk of Incident NAFLD According to PRS and Biological Aging

**Figure 2:**
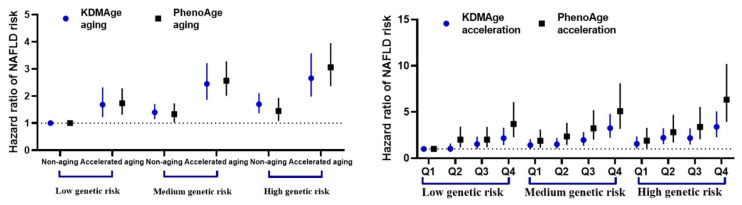
Joint effects of biological aging and the PRS on the risk of incident NAFLD in UK Biobank. Models were adjusted for age, sex, body mass index, ethnicity, smoking status, alcohol intake, metabolic equivalent task, and education level. Abbreviations: NAFLD, Non-alcoholic fatty liver disease; PRS, polygenic risk score; Q, quantile.

A high PRS was positively associated with the risk of incident NAFLD; the HR (95% CI) was 2.15 (1.89, 2.44) (Table S5). When stratified by genetic risk, accelerated aging individuals also exhibited an increased risk of NAFLD regardless of genetic risk. The HRs (95% CI) for KDMAge were 1.36 (0.86, 2.16), 1.91 (1.42, 2.57), and 1.53 (1.03, 2.26) and for PhenoAge were 1.69 (1.27, 2.26), 1.96 (1.65, 2.32), and 2.08 (1.65, 2.62) in the low-genetic-risk, medium-genetic-risk, and high-genetic-risk populations, respectively (Table 3). In our study, no significant interaction between biological aging and PRS was observed in relation to the risk of NAFLD (Table S6). However, we found a joint effect of biological aging and PRS on the risk of incident NAFLD. The highest risk of developing NAFLD was observed in individuals with high genetic predisposition and accelerated aging, as opposed to those with low genetic predisposition and non-aging [KDMAge: 2.66 (1.98, 3.57); PhenoAge: 3.05 (2.36, 3.95)]. In addition, individuals who had a high genetic risk and were in Q4 of KDMAge/PhenoAge acceleration exhibited the highest risk of incident NAFLD when compared to those with a low PRS plus Q1 [KDMAge: 3.39 (2.27, 5.07); PhenoAge: 6.33 (3.93, 10.20)] (Figure 2).

## 4. Discussion

Based on using clinical biomarkers to calculate the age-adjusted biological age in both the UK Biobank and Dongfeng–Tongji Cohort, our research revealed that accelerated biological aging was positively associated with the risk of incident NAFLD. Participants with high genetic risk and accelerated aging had a significantly higher risk of incident NAFLD compared with participants with low genetic risk and non-aging individuals. However, no significant interaction between genetic risk and biological aging was observed in relation to NAFLD risk, indicating biological aging was associated with the risk of NAFLD regardless of genetic risk.

In the current study, we investigated the association between biological aging and NAFLD incidence from different disease processes and different ethnicities. In the UK Biobank, identifying NAFLD through hospital records and death certificates may indeed lead to selection bias, as it predominantly includes more severe cases. However, despite the limitations of the diagnostic methods in the UK Biobank, its long-term follow-up and large sample size provide stable data to assess the association between biological aging and more severe NAFLD. Meanwhile, the Dongfeng–Tongji Cohort used ultrasound diagnostics, which can detect cases in the early stages of the disease. The combination of both datasets allows for the validation of the association between biological aging and NAFLD across different disease stages, making the findings more persuasive. Additionally, the consistency of results between the two cohort studies further supports the robustness of the conclusions. In our study, referring to previous research, the indicators used to calculate KDMAge in both cohorts were based on their strong correlation with chronological age [24,26,27]. The selected biomarkers may vary slightly due to differences in populations, sample sizes, and the availability of testing indicators. However, the same conclusions were obtained in both cohorts, which further supports the robustness of our results.

Recent studies have also found associations between biological aging and NAFLD. For instance, recent studies have reported the inverse associations of telomere length with NAFLD risk [28,29]. However, this single indicator provides limited insights into organ or systemic aging. Previous studies showed that KDMAge and PhenoAge were favored in terms of their superior ability in predicting age-related outcomes, such as diabetes, osteoarthritis, and depression [26,30,31]. Together, KDMAge and PhenoAge could be employed as a comprehensive and easily accessible tool to identify individuals experiencing biological aging and to prevent the onset of NAFLD. In addition, recent studies conducted by Xia et al. have emphasized the role of DNA methylation age acceleration in NAFLD development, providing further insight into the role of biological aging in NAFLD [32]. Wang et al. showed that biological aging is linked to an increased risk of mortality in patients with NAFLD, reinforcing the robustness of aging metrics in predicting liver disease trajectories [13]. These studies collectively underscore the translational potential of biological aging biomarkers in NAFLD.

Animal studies have shown that biological aging was involved in the pathogenesis of NAFLD. Existing research has found a close relationship between aging markers and fat accumulation in the liver cells of mice. Cell senescence can autonomously lead to steatosis by triggering mitochondrial dysfunction, which impairs fat metabolism [33]. A recent study demonstrated that senescent cells in the liver secrete pro-inflammatory cytokines that disrupt normal liver function and promote the development of NAFLD [34]. This finding provides additional mechanistic evidence for the link between biological aging and NAFLD. Deficiency in hepatic peroxisome proliferator-activated receptor alpha (PPARα) results in reduced fatty acid β-oxidation in the liver [35]. Montagner et al. found that specific depletion of PPARα in hepatocytes can cause lipid accumulation in the liver and exacerbate NAFLD in aging mice, mediated through the regulation of fibroblast growth factor 21 (FGF21) [36]. In addition, the regulation of aging-related genes can alter intrahepatic fat deposition and modulate the risk of developing NAFLD [9,10]. The above research consistently supported the relationship between accelerated aging and NAFLD. Accumulating evidence indicated that lifestyle (e.g., Mediterranean diet and intermittent fasting) and pharmacological (e.g., metformin) interventions can mitigate the risk of aging-related diseases [37,38,39]. Clinical trials have shown that these interventions may specifically benefit NAFLD patients by reducing biological age. For example, several previous studies have shown that the Mediterranean diet is associated with a reduction in the prevalence and severity of NAFLD [40,41]. These interventions may delay biological aging by modulating metabolism, alleviating oxidative stress, and ameliorating the inflammatory response. Considering the potential link between biological aging and NAFLD risk, future studies could explore whether delaying biological aging can lower the risk of NAFLD. Specifically, clinical trials on these interventions can offer valuable insights into the relationship between biological aging and NAFLD, facilitating the application of anti-aging early-intervention strategies in NAFLD prevention.

Compelling evidence has demonstrated that genetic susceptibility is responsible for NAFLD risk [42,43], which is consistent with our findings. We further evaluated the potential interaction between biological aging and the genetic risk of NAFLD. In our study, no significant interaction between genetic risk and biological aging was observed in relation to NAFLD risk. This suggested that these factors may affect NAFLD via different molecular pathways. Genetic variants influence lipid metabolism and insulin resistance [44,45,46], while biological aging reflects systemic changes in organ function and inflammation [47]. Additionally, epigenetic modifications associated with biological aging may modulate gene expression independently of genetic risk [48], further explaining the lack of interaction. The absence of interaction could also be influenced by statistical power. The SNP we used to assess genetic risk is based on known ones, but there may be other unknown genetic variants not included in the analysis. Moreover, indicators for calculating biological aging rely on a set of biomarkers, which may not fully reflect the complexity of the biological aging process. Future studies with comprehensive biomarkers of biological aging, as well as more granular genetic analyses, may provide deeper insights into potential interactions.

A key strength of this study is its use of general population data from two well-established cohorts in the UK and China, with results being largely consistent across both cohorts. However, several notable limitations should be acknowledged. First, while we accounted for many relevant confounding lifestyle factors, some potential influencing factors, such as dietary structure, socioeconomic factors, psychological factors, environmental exposures, and other residual confounding, cannot be excluded. These unmeasured factors have the potential to distort the true associations between biological aging, genetic risk, and NAFLD. Future research that incorporates these elements will be crucial for enhancing the reliability of study conclusions. Second, lifestyle factors like alcohol intake were assessed through questionnaires rather than objective alcohol markers, which could introduce recall or misclassification bias. Thirdly, most biological aging metrics were only measured at baseline. Given that biological aging is a dynamic process, the absence of longitudinal data on these markers significantly restricts our ability to explore the time-varying changes in biological aging and their relationship with NAFLD development. Longitudinal studies with repeated measurements of these biomarkers are essential to fully understand this temporal relationship. Fourthly, our sample primarily consisted of middle-aged and elderly volunteers at baseline. This demographic limitation raises concerns about the generalizability of our findings to younger populations, as well as those from different ethnic, cultural, and socioeconomic backgrounds. Younger individuals may have distinct biological aging trajectories and NAFLD risk profiles, and populations with diverse genetic and environmental exposures may exhibit different associations. Future research should aim to include more diverse samples to improve the external validity of the results. Fifth, the SNPs included in our polygenic risk score were derived from European populations. The generalizability of our findings may be influenced by the genetic ancestry of the GWAS data used to construct the polygenic risk score. Developing and validating polygenic risk scores in diverse populations will be necessary to enhance the accuracy of genetic risk prediction for NAFLD. Finally, it is important to highlight that our study was observational in nature. As such, establishing a definitive causal link between biological aging, genetic risk, and NAFLD remains challenging. Observational studies are prone to issues such as residual confounding and reverse causation, which can obscure the true nature of these associations. To confirm the causal relationships suggested by our findings, experimental studies, such as randomized controlled trials with interventions targeting biological aging or genetic expression, are required. These limitations underscore the need for further research to fully elucidate the complex relationships between biological aging, genetic factors, and NAFLD.

Collectively, both biological aging and high genetic risk were significantly associated with higher NAFLD risk. The association between biological age and NAFLD was independent of genetic risk of NAFLD.

## 5. Conclusions

Our study demonstrated that accelerated biological aging was associated with an increased risk of incident NAFLD, with effects persisting regardless of genetic susceptibility. Given the significant association between biological aging and NAFLD, further elucidating the mechanistic links between biological aging processes and hepatic steatosis, along with developing targeted interventions to modulate biological aging, is crucial for translating these epidemiological findings into effective NAFLD prevention strategies.

## Figures and Tables

**Table 1:** Comparisons of baseline characteristics between NAFLD and non-NAFLD participants from two cohorts.

	UK Biobank			Dongfeng–Tongji Cohort		
Characteristics	Total (N = 329,040)	Non-NAFLD (N = 325,266)	NAFLD (N = 3774)	Total (N = 6783)	Non-NAFLD (N = 4985)	NAFLD (N = 1798)
Age, years	56.4 ± 8.1	56.4 ± 8.1	56.8 ± 8.0	62.0 ± 7.4	62.5 ± 7.4	60.6 ± 7.3
Sex (%)						
Female	177,498 (53.9)	175,556 (54.0)	1942 (51.5)	3969 (58.5)	2710 (54.4)	1259 (70.1)
Male	151,542 (46.1)	149,710 (46.0)	1832 (48.5)	2810 (41.5)	2272 (45.6)	538 (29.9)
Race/ethnicity (%)						
Non-White	16,619 (5.1)	16,372 (5.1)	247 (6.6)	-	-	-
White	310,927 (94.6)	307,420 (94.6)	3507 (93.0)	-	-	-
BMI (%)						
<25 or 24	109,867 (33.5)	109,504 (33.7)	363 (9.7)	4156 (61.3)	3330 (66.8)	826 (46.0)
25–30 or 24–28	140,526 (42.8)	139,137 (42.9)	1389 (37.0)	2274 (33.5)	1463 (29.4)	811 (45.1)
≥30 or 28	77,959 (23.7)	75,955 (23.4)	2004 (53.3)	351 (5.2)	191 (3.8)	160 (8.9)
Smoking status (%)						
Never	180,864 (55.0)	179,172 (55.1)	1692(44.9)	5056 (74.9)	3598 (72.5)	1458(81.4)
Ever	113,278 (34.5)	111,773 (34.4)	1505 (40.0)	686 (10.2)	550 (11.1)	136 (7.6)
Current	33,351 (10.0)	32,797 (10.1)	554 (14.7)	1012 (15.0)	815 (16.4)	197 (11.0)
Alcohol intake (%)						
Never	14,071 (4.3)	13,842 (4.2)	229 (6.1)	5621 (77.6)	3813 (76.5)	1448 (80.5)
Ever	11,050 (3.4)	10,796 (3.3)	254 (6.8)	358 (5.3)	296 (6.0)	62 (3.5)
Current	303,190 (92.2)	299,917 (92.3)	3273 (86.8)	1162 (17.1)	874 (17.5)	288(16.0)
Education level (%)						
Less than high school	89,372 (33.0)	88,261 (32.9)	1111 (39.8)	4416 (65.1)	3239 (65.5)	1177 (65.0)
High school or above	181,870 (67.0)	180,191 (67.1)	1679 (60.2)	2367 (34.9)	1746 (34.5)	621 (35.0)
Metabolic equivalent task, min/week	1785.0 (813.0, 3573.0)	1786.0 (815.0, 3573.0)	1480.0 (611.0, 3230.0)	1674.0 (1080.0, 2700.0)	1680.0 (1050.0, 2744.0)	1674.0 (1080.0, 2520.0)
KDMAge, years	61.0 (54.0, 67.0)	61.0 (54.0, 67.0)	63.0 (56.0, 68.0)	53.0 (38.0, 71.0)	53.0 (37.0, 71.0)	54.0 (39.0, 71.0)
PhenoAge, years	46.0 (38.0, 53.0)	46.0 (38.0, 53.0)	49.0 (42.0, 56.0)	-	-	-
KDMAge, non-aging (%)	223,635 (68.0)	221,375 (68.1)	2260 (31.9)	4380 (64.0)	3267 (64.8)	1112 (61.7)
KDMAge, accelerated aging (%)	105,405 (32.0)	103,891 (59.9)	1514 (40.1)	2403 (36.0)	1717 (35.2)	686 (38.3)
PhenoAge, non-aging (%)	179,531 (54.6)	178,257 (54.8)	1274 (33.8)	-	-	-
PhenoAge, accelerated aging (%)	149,509 (45.4)	147,009 (45.2)	2500 (66.2)	-	-	-

Data were presented as frequency (%), mean (standard deviation), or median (interquartile range). Non-aging: KDMAge/PhenoAge acceleration ≤ 0. Accelerated aging: KDMAge/PhenoAge acceleration > 0. Abbreviations: NAFLD, non-alcoholic fatty liver disease; BMI, body mass index.

**Table 2:** Risk of incident NAFLD according to biological aging in two cohorts.

	UK Biobank				Dongfeng–Tongji Cohort	
	Hazard Ratio (95% CI)	*p*-Value	Hazard Ratio (95% CI)	*p*-Value	Odds Ratio (95% CI)	*p*-Value
Case/total	3774/325,266				1798/4985	
	KDMAge acceleration		PhenoAge acceleration		KDMAge acceleration	
Continuous	1.06 (1.04, 1.07)	<0.001	1.06 (1.05, 1.06)	<0.001	1.01 (1.00, 1.01)	0.001
Q1	1 (Reference)		1 (Reference)		1 (Reference)	
Q2	1.19 (1.04, 1.37)	0.015	1.25 (1.07, 1.45)	0.005	1.34 (1.13, 1.60)	0.001
Q3	1.34 (1.16, 1.55)	<0.001	1.44 (1.24, 1.67)	<0.001	1.41 (1.18, 1,69)	<0.001
Q4	1.76 (1.49, 2.09)	<0.001	2.38 (2.06, 2.74)	<0.001	1.50 (1.24, 1.83)	<0.001
*p* for trend		<0.001		<0.001		<0.001
Non-aging	1 (Reference)		1 (Reference)		1 (Reference)	0.019
Accelerated aging	1.35 (1.17, 1.56)	<0.001	1.69 (1.54, 1.85)	<0.001	1.18 (1.03, 1.36)	

Models were adjusted for age, sex, body mass index, ethnicity (in the UK Biobank), smoking status, alcohol intake, metabolic equivalent task, and education level. Non-aging: KDMAge/PhenoAge acceleration ≤ 0. Accelerated aging: KDMAge/PhenoAge acceleration > 0. Abbreviations: NAFLD, non-alcoholic fatty liver disease; Q, quantile.

**Table 3:** Hazard ratio for NAFLD risk based on biological aging, stratified by PRS in the UK Biobank.

	Low Genetic Risk		Medium Genetic Risk		High Genetic Risk	
	Hazard Ratio (95% CI)	*p*-Value	Hazard Ratio (95% CI)	*p*-Value	Hazard Ratio (95% CI)	*p*-Value
Case/total	404/50,796		1057/101,697		623/50,857	
KDMAge acceleration						
Continuous	1.05 (1.01, 1.10)	0.008	1.09 (1.07, 1.11)	<0.001	1.05 (1.02, 1.09)	0.001
Q1	1 (Reference)		1 (Reference)		1 (Reference)	
Q2	1.06 (0.67, 1.65)	0.816	1.02 (0.78, 1.33)	0.906	1.52 (1.08, 2.14)	0.016
Q3	1.51 (0.96, 2.37)	0.076	1.34 (1.02, 1.77)	0.036	1.51 (1.04, 2.19)	0.030
Q4	1.91 (1.13, 3.25)	0.017	2.55 (1.83, 3.56)	<0.001	2.01 (1.30, 3.12)	0.002
*p* for trend	0.011		<0.001		0.003	
Non-aging	1 (Reference)		1 (Reference)		1 (Reference)	
Accelerated aging	1.36 (0.86, 2.16)	0.193	1.91 (1.42, 2.57)	<0.001	1.53 (1.03, 2.26)	0.034
PhenoAge acceleration						
Continuous	1.06 (1.04, 1.08)	<0.001	1.06 (1.05, 1.07)	<0.001	1.07 (1.05, 1.08)	<0.001
Q1	1 (Reference)		1 (Reference)		1 (Reference)	
Q2	1.94 (1.14, 3.33)	0.015	1.27 (0.94, 1.71)	0.118	1.47 (0.98, 2.22)	0.064
Q3	1.93 (1.13, 3.28)	0.016	1.75 (1.32, 2.32)	<0.001	1.76 (1.18, 2.61)	0.005
Q4	3.60 (2.17, 5.97)	<0.001	2.75 (2.10, 3.61)	<0.001	3.26 (2.24, 4.73)	<0.001
*p* for trend	<0.001		<0.001		<0.001	
Non-aging	1 (Reference)		1 (Reference)		1 (Reference)	
Accelerated aging	1.69 (1.27, 2.26)	<0.001	1.96 (1.65, 2.32)	<0.001	2.08 (1.65, 2.62)	<0.001

Models were adjusted for age, sex, BMI, ethnicity, smoking status, alcohol intake, metabolic equivalent task, education level, genotyping batch, and genetic principal components. Non-aging: KDMAge/PhenoAge acceleration ≤ 0. Accelerated aging: KDMAge/PhenoAge acceleration > 0. Abbreviations: NAFLD, non-alcoholic fatty liver disease; PRS, polygenic risk score; Q, quantile.

## Data Availability

The UK Biobank resource can be accessed by researchers on application. Data are available from the UK Biobank (https://www.ukbiobank.ac.uk/, accessed on 1 January 2024). For the Dongfeng–Tongji Cohort, access to the dataset is subject to approval by the Dongfeng–Tongji Cohort management committee. Researchers interested in accessing the data may submit a formal request to the committee outlining the purpose and scope of their research. Upon approval, the committee will provide access to the data for the approved research purposes.

## Supplementary Materials

The following supporting information can be downloaded at https://www.mdpi.com/article/10.3390/nu17101618/s1; Figure S1: The flow chart of the participants at baseline from UK Biobank; Figure S2: The flow chart of the participants at baseline from Dongfeng-Tongji Cohort study; Figure S3: Stratified analyses of the associations between biological aging and the risk of prevalent and incident NAFLD; Table S1: Hazard ratio of NAFLD risk based on biological aging after excluding NAFLD cases that occurred in the first two-year of follow-up in UK Biobank; Table S2: Hazard ratio or Odds ratio of NAFLD risk based on biological aging after further adjusting for liver disease related covariates in two cohorts; Table S3: Hazard ratio or odds ratio of NAFLD risk based on biological aging after filling in the missing values in two cohorts; Table S4: Hazard ratio or odds ratio NAFLD risk based on biological aging after further adjusting the participant’s illness status in two cohorts; Table S5: Risk of incident NAFLD according to genetic risk in UK Biobank; Table S6: Additive and multiplicative interactions between biological aging and PRS on risk of incident NAFLD in UK Biobank; Table S7: Single-nucleotide polymorphisms used to build the genetic risk score for NAFLD. Reference [49] is cited in Supplementary Materials.
