# Supplementary material for: Accelerated Biological Aging, Genetic Susceptibility, and Non-Alcoholic Fatty Liver Disease: Two Prospective Cohort Studies
Source: Nutrients. 2025 May 8;17(10):1618. doi: 10.3390/nu17101618 (PMC12113898; doi:10.3390/nu17101618)
Supplement: Supplementary file 1 [file nutrients-17-01618-s001.zip › nutrients-3616695-supplementary.pdf]

Supplementary materials

Figures:

Figure S1. The flow chart of the participants at baseline from UK Biobank.

Figure S2. The flow chart of the participants at baseline from Dongfeng-Tongji Cohort study.

Figure S3. Stratified analyses of the associations between biological aging and the risk of prevalent and incident NAFLD.

Tables:

Table S1. Hazard ratio of NAFLD risk based on biological aging after excluding NAFLD cases that occurred in the first two-year of follow-up in UK Biobank.

Table S2. Hazard ratio or Odds ratio of NAFLD risk based on biological aging after further adjusting for liver disease related covariates in two cohorts.

Table S3. Hazard ratio or odds ratio of NAFLD risk based on biological aging after filling in the missing values in two cohorts.

Table S4 Hazard ratio or odds ratio NAFLD risk based on biological aging after further adjusting the participant's illness status in two cohorts.

Table S5. Risk of incident NAFLD according to genetic risk in UK Biobank.

Table S6 Additive and multiplicative interactions between biological aging and PRS on risk of incident NAFLD in UK Biobank.

Table S7. Single-nucleotide polymorphisms used to build the genetic risk score for NAFLD.

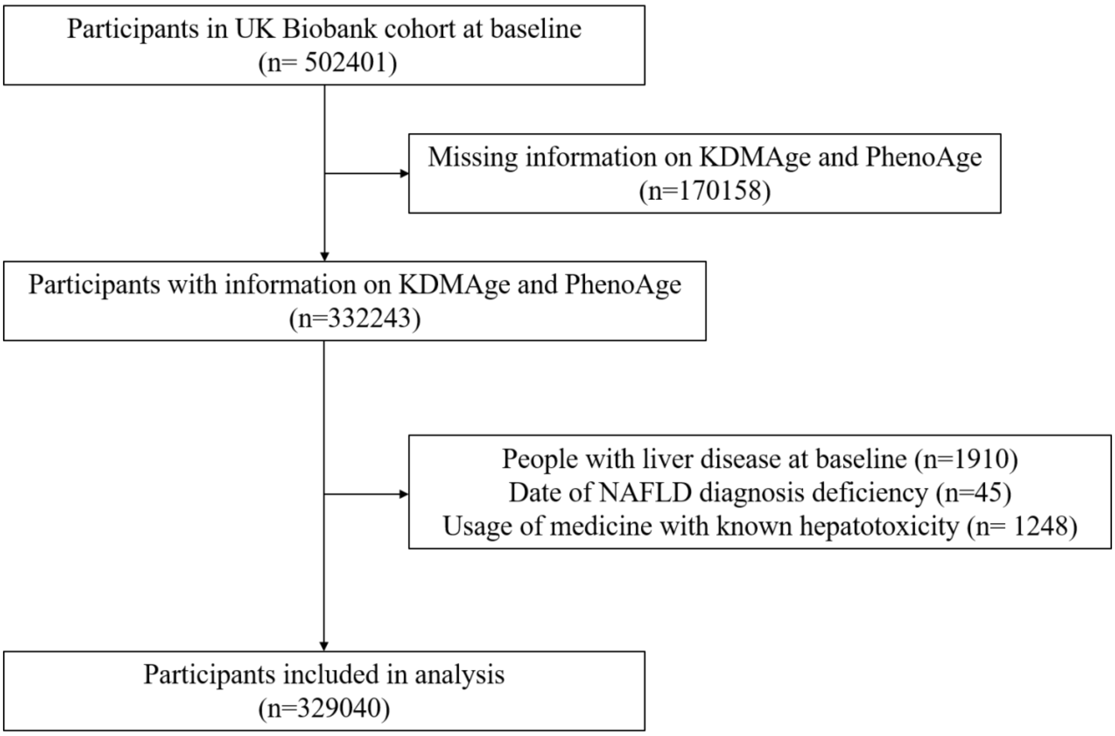

Figure S1. The flow chart of the participants at baseline from UK Biobank.

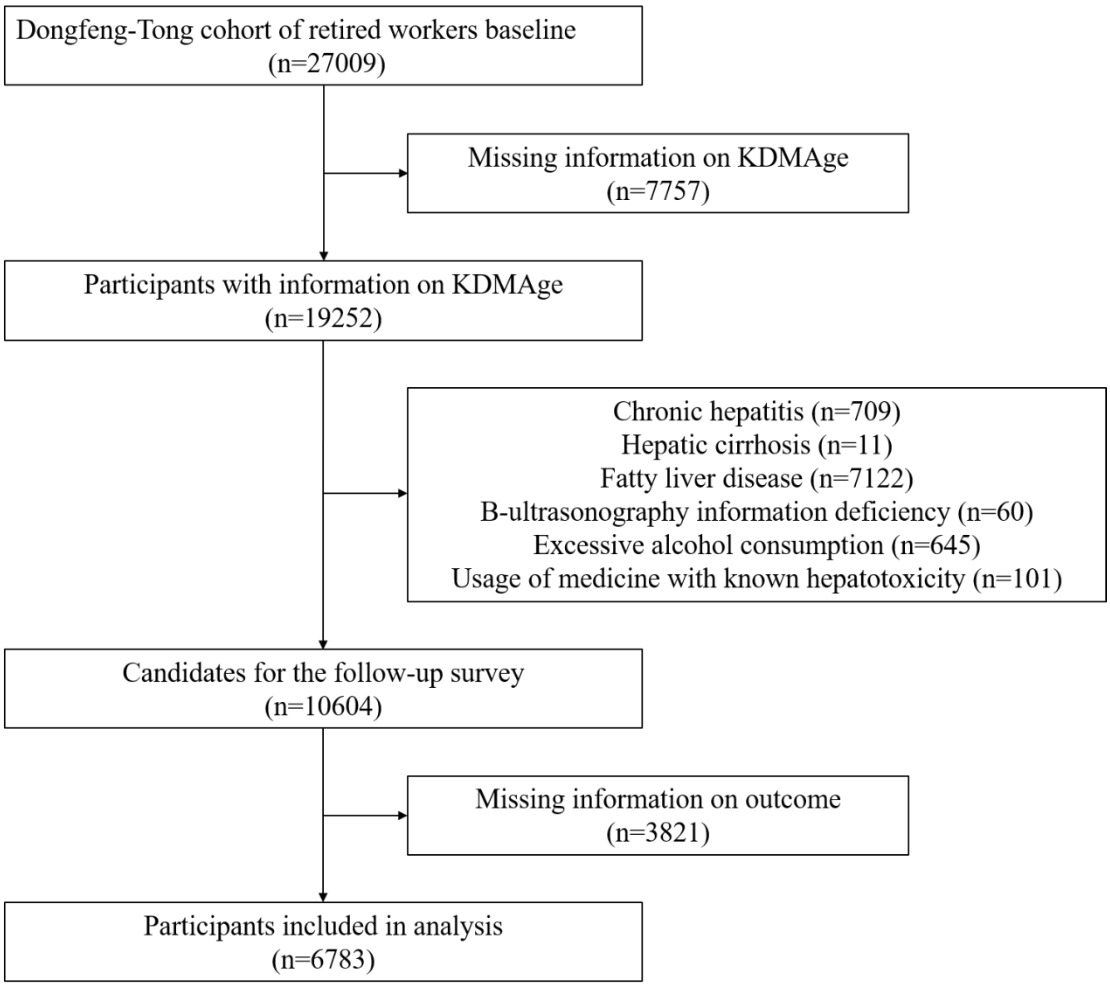

Figure S2. The flow chart of the participants at baseline from Dongfeng-Tongji Cohort study.

Table S1. Hazard ratio of NAFLD risk based on biological aging after excluding NAFLD cases that occurred in the first two-year of follow-up in UK Biobank.

	**Hazard ratio (95% CI)**	***P*-value**	**Hazard ratio (95% CI)**	***P*-value**
Case/total	3588/323800			
	KDMAge acceleration		PhenoAge acceleration	
Continuous	1.05 (1.04, 1.07)	<0.001	1.06 (1.05, 1.06)	<0.001
Q1	1 (Reference)		1 (Reference)	
Q2	1.17 (1.01, 1.35)	0.037	1.22 (1.04, 1.43)	0.013
Q3	1.33 (1.14, 1,54)	<0.001	1.42 (1.22, 1.65)	<0.001
Q4	1.73 (1.46, 2.05)	<0.001	2.32 (2.01, 2.68)	<0.001
P for trend	<0.001		<0.001	
Non-aging	1 (Reference)		1 (Reference)	
Accelerated aging	1.34 (1.15, 1.56)	<0.001	1.69(1.54, 1.85)	<0.001

Models were adjusted for age, sex, body mass index, ethnicity, smoking status, alcohol intake, metabolic equivalent task, and education level. Non-aging: KDMAge/PhenoAge acceleration ≤0. Accelerated aging: KDMAge/PhenoAge acceleration >0. Abbreviations: NAFLD, Non-alcoholic fatty liver disease; Q, quantile.

Table S2. Hazard ratio or odds ratio of NAFLD risk based on biological aging after further adjusting for liver disease related covariates in two cohorts.

	**UK Biobank**				**Dongfeng-Tongji cohort**	
	Hazard ratio (95% CI)	*P*-value	Hazard ratio (95% CI)	*P*-value	Odds ratio (95% CI)	*P*-value
Case/total	3774/325266				1798/4985	
	KDMAge acceleration		PhenoAge acceleration		KDMAge acceleration	
Continuous	1.05 (1.04, 1.06)	<0.001	1.05 (1.05, 1.06)	<0.001	1.01 (1.00, 1.01)	0.001
Q1	1 (Reference)		1 (Reference)		1 (Reference)	
Q2	1.14 (0.99, 1.31)	0.073	1.23 (1.05, 1.44)	0.009	1.33 (1.12, 1.58)	0.001
Q3	1.20 (1.03, 1,38)	0.017	1.43 (1.23, 1.66)	<0.001	1.41(1.17, 1,69)	<0.001
Q4	1.53 (1.29, 1.81)	<0.001	2.28 (1.98, 2.63)	<0.001	1.51 (1.24, 1.84)	<0.001
P for trend	<0.001		<0.001		<0.001	
Non-aging	1 (Reference)		1 (Reference)		1 (Reference)	
Accelerated aging	1.17 (1.02, 1.35)	0.029	1.65 (1.50, 1.80)	<0.001	1.19(1.03, 1.37)	0.017

Models were adjusted for age, sex, body mass index, ethnicity (in the UK Biobank), smoking status, alcohol intake, metabolic equivalent task, education level, ALT, AST and TBIL. Non-aging: KDMAge/PhenoAge acceleration ≤0. Accelerated aging: KDMAge/PhenoAge acceleration >0. Abbreviations: NAFLD, Non-alcoholic fatty liver disease; Q, quantile; ALT, glutamic-pyruvic transaminase; AST, glutamic oxaloacetic transaminase; TBIL, total bilirubin.

Table S3. Hazard ratio or odds ratio of NAFLD risk based on biological aging after filling in the missing values in two cohorts.

	**UK Biobank**				**Dongfeng-Tongji cohort**	
	Hazard ratio (95% CI)	*P*-value	Hazard ratio (95% CI)	*P*-value	Odds ratio (95% CI)	*P*-value
Case/total	1176/298213				1176/298213	
	KDMAge acceleration		PhenoAge acceleration		KDMAge acceleration	
Continuous	1.05 (1.04, 1.06)	<0.001	1.05 (1.05, 1.06)	<0.001	1.01 (1.00, 1.01)	<0.001
Q1	1 (Reference)		1 (Reference)		1 (Reference)	
Q2	1.19 (1.06, 1.33)	0.003	1.24 (1.10, 1.40)	0.001	1.32(1.11, 1.55)	0.001
Q3	1.31 (1.17, 1,47)	<0.001	1.48 (1.32, 1.66)	<0.001	1.41(1.18, 1,68)	<0.001
Q4	1.65 (1.44, 1.88)	<0.001	2.13 (1.90, 2.37)	<0.001	1.56(1.27, 1.92)	<0.001
P for trend	<0.001		<0.001			<0.001
Non-aging	1 (Reference)		1 (Reference)		1 (Reference)	
Accelerated aging	1.30 (1.16, 1.45)	<0.001	1.62 (1.51, 1.73)	<0.001	1.20 (1.04, 1.38)	0.014

Models were adjusted for age, sex, body mass index, ethnicity, smoking status, alcohol intake, metabolic equivalent task, and education level. Non-aging: KDMAge/PhenoAge acceleration ≤0. Accelerated aging: KDMAge/PhenoAge acceleration >0. Abbreviations: NAFLD, Non-alcoholic fatty liver disease; Q, quantile.

Table S4. Hazard ratio or odds ratio NAFLD risk based on biological aging after further adjusting the participant's illness status in two cohorts.

	**UK Biobank**				**Dongfeng-Tongji cohort**	
	Hazard ratio (95% CI)	*P*-value	Hazard ratio (95% CI)	*P*-value	Odds ratio (95% CI)	*P*-value
Case/total	1176/298213				1798/4985	
	KDMAge acceleration		PhenoAge acceleration		KDMAge acceleration	
Continuous	1.03 (1.04, 1.06)	<0.001	1.06 (1.05, 1.06)	<0.001	1.00 (1.00, 1.01)	0.018
Q1	1 (Reference)		1 (Reference)		1 (Reference)	
Q2	1.18 (0.96, 1.45)	0.127	1.22 (1.01, 1.48)	0.040	1.30 (1.09, 1.55)	0.004
Q3	1.18 (0.95, 1,46)	0.137	1.46 (1.22, 1.76)	<0.001	1.37(1.13, 1,66)	0.001
Q4	1.45 (1.14, 1.83)	0.002	2.37 (1.99, 2.81)	<0.001	1.43 (1.15, 1.77)	0.001
P for trend	0.005		<0.001			0.001
Non-aging	1 (Reference)		1 (Reference)		1 (Reference)	
Accelerated aging	1.26 (1.04, 1.52)	0.019	1.66 (1.49, 1.86)	<0.001	1.13 (0.98, 1.32)	0.102

Models were adjusted for age, sex, body mass index, ethnicity, smoking status, alcohol intake, metabolic equivalent task, education level, hypertension, diabetes, hyperlipidemia and cancer. Non-aging: KDMAge/PhenoAge acceleration ≤0. Accelerated aging: KDMAge/PhenoAge acceleration >0. Abbreviations: NAFLD, Non-alcoholic fatty liver disease; Q, quantile.

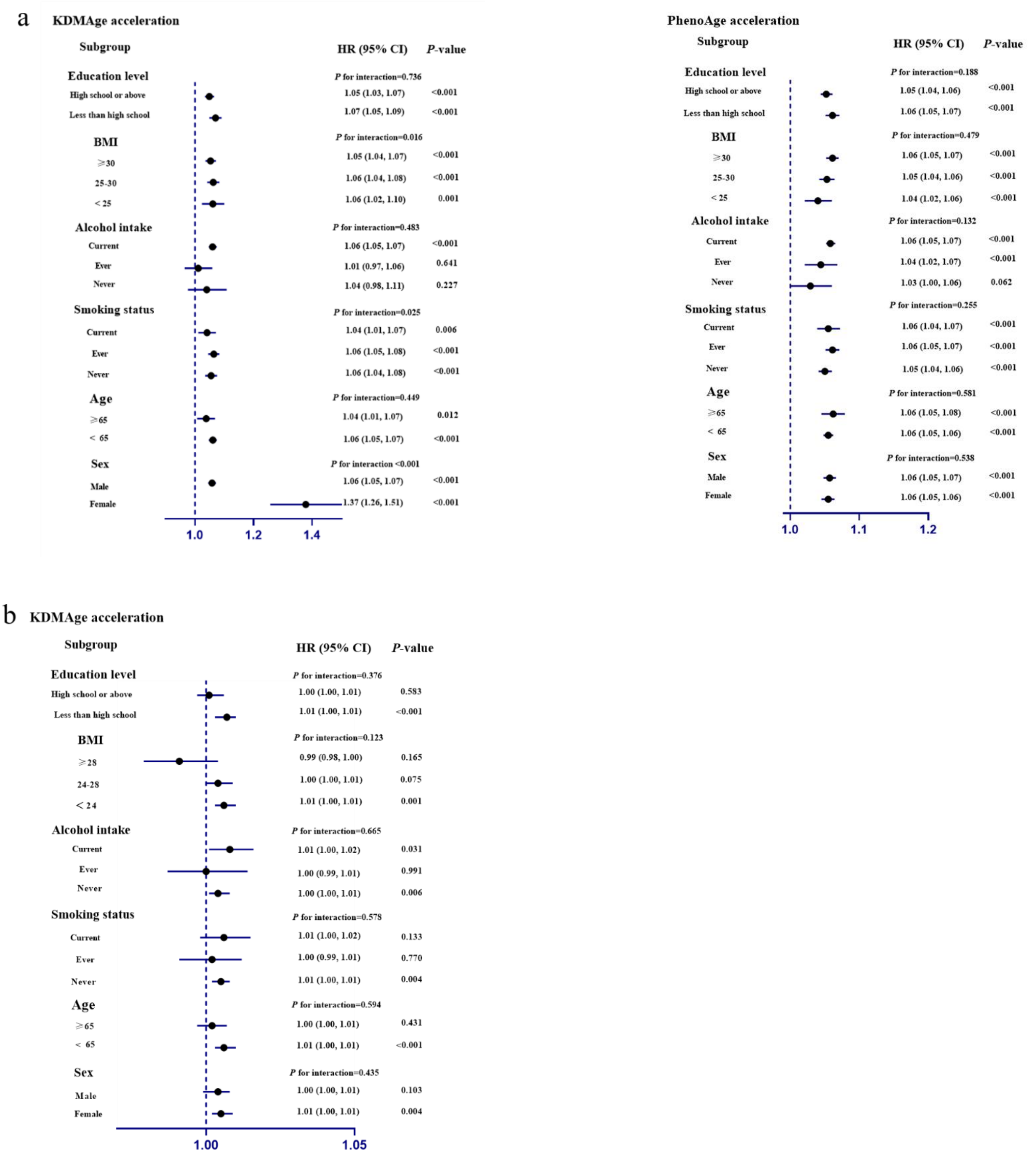

Figure S3. Hazard ratio/Odds ratio of NAFLD risk based on biological aging in two cohorts stratified by sex, age, BMI, smoking status, alcohol intake and education level (a, UK Biobank; b, Dongfeng-Tongji Cohort). Models were adjusted for age, sex, body mass index, ethnicity (in the UK Biobank), smoking status, alcohol intake, metabolic equivalent task, education level.

Table S5. Risk of incident NAFLD according to genetic risk in UK Biobank.

	**Continuous**	**Low genetic risk**	**Medium genetic risk**	**High genetic risk**	***P* for trend**
Case/total	3774/325266	404/50392	1057/100640	623/50234	
HR (95% CI)	2.15 (1.89, 2.44)	1 (Reference)	1.43 (1.22, 1.66)	1.65 (1.40, 1.95)	
P-value	<0.001		<0.001	<0.001	<0.001

Models were adjusted for age, sex, body mass index, ethnicity, smoking status, alcohol intake, metabolic equivalent task, education level, genotyping batch, and genetic principal components. Abbreviations: NAFLD, Non-alcoholic fatty liver disease.

Table S6. Additive and multiplicative interactions between biological aging and PRS on risk of incident NAFLD in UK Biobank.

	RERI (95% CI)	AP (95% CI)	*P*-value additive interactions	*P*-value multiplicative interactions
KDMAge acceleration				
Continuous	0.05 (-0.01, 0.11)	0.02 (-0.01, 0.05)	0.107	0.478
Non-aging- Accelerated aging	0.27 (-0.45, 1.00)	0.10 (-0.13, 0.32)	0.460	0.602
PheoAge acceleration				
Continuous	0.03 (-0.01, 0.07)	0.02 (-0.01, 0.04)	0.089	0.110
Non-aging- Accelerated aging	0.29 (-0.50, 1.09)	0.08 (-0.13, 0.30)	0.470	0.113

Models were adjusted for age, sex, body mass index, ethnicity, smoking status, alcohol intake, metabolic equivalent task, education level, genotyping batch, and genetic principal components. Non-aging: KDMAge/PhenoAge acceleration ≤0. Accelerated aging: KDMAge/PhenoAge acceleration > 0. Abbreviations: NAFLD, Non-alcoholic fatty liver disease; PRS, polygenic risk score; RERI, relative excess risk due to interaction; AP, attributable proportion due to interaction.

Table S7. Single-nucleotide polymorphisms used to build the genetic risk score for NAFLD.

SNP	Nominated Gene	EA/NEA	β
Main analysis			
rs79598313	SYTL1; GPN2; PIGV; KDF1; lnc-SFN-1	T/C	0.180
rs1337101	SLC30A10	T/G	-0.051
rs2642438	MTARC1	A/G	-0.079
rs848559	CRIM1	T/A	-0.061
rs10195619	MERTK	T/C	-0.049
rs13409360	IL1RN	A/G	-0.060
rs73024760	ABCB11	T/C	0.107
rs10201587	CASP8	G/A	-0.045
rs2138157	MIR5702; lnc-RHBDD1-6; lnc-RHBDD1-3	A/C	-0.064
rs7604422	EFHD1	C/A	-0.053
rs4684847	PPARG	T/C	-0.072
rs9867368	PCCB	A/G	-0.078
rs12486792	SLC2A2	G/C	0.060
rs12500824	SHROOM3	A/G	0.046
rs4841133	PPP1R3B	A/G	0.130
rs4484649	SOX7; RP1L1; C8orf74	C/A	0.045
rs4734654	KLF10	G/A	-0.052
rs2954038	lnc-TRIB1-2; WASHC5	C/A	0.139
rs7041363	AKNA	G/C	-0.135
rs17780834	FBXL15; ELOVL3; PSD	T/A	0.086
rs2792751	GPAM	T/C	0.072
rs174535	FADS2	C/T	-0.061
rs7117339	PANX1	T/C	-0.130
rs4919741	KRT84; KRT82; KRT74	A/G	-0.057
rs1169292	HNF1A (P2RX7)	T/C	0.054
rs148015593	MLXIP	G/T	-0.042
rs28929474	SERPINA1	T/C	0.481
rs168144	RORA	C/T	-0.052
rs55868793	CD276	G/T	0.059
rs72754571	ANPEP	A/C	-0.083
rs112128680	DHODH; HP	A/G	0.061
rs1801689	APOH	C/A	0.176
rs4940689	NEDD4L	A/G	0.054
rs3810367	STAP2; MPND	G/T	0.045
rs58542926	TM6SF2	T/C	0.222
rs7599	TMEM147; ATP4A	A/G	0.049
rs429358	APOE; APOC1	C/T	-0.094
rs2377957	ITCH	A/G	-0.053
rs2207132	MAFB	A/G	0.189
rs1547014	CHEK2	T/C	-0.064
rs132665	APOL3	G/A	-0.069
rs738409	PNPLA3	G/C	0.269
Additional analysis			
rs641738	MBOAT7-TMC4 [1]	T/C	0.166

SNP, single nucleotide polymorphism; EA, effect allele; NEA, non-effect allele.

References:
[1]. Mancina RM, Dongiovanni P, Petta S, et al. The MBOAT7-TMC4 Variant rs641738 Increases Risk of Nonalcoholic Fatty Liver Disease in Individuals of

European Descent. Gastroenterology. 2016;150(5):1219-1230.e6
